# Redefining the concept of resectability in the landscape of perioperative treatments for lung cancer

**DOI:** 10.3389/fonc.2026.1903430

**Published:** 2026-07-22

**Authors:** Francesco Petrella, Andrea Cara, Enrico Mario Cassina, Jessica Dell’Agosto, Lidia Libretti, Emanuele Pirondini, Federico Raveglia, Antonio Tuoro

**Affiliations:** 1 Department of Thoracic Surgery, Fondazione Istituto di Ricovero e Cura a Carattere Scientifico (IRCCS) San Gerardo dei Tintori, Monza, Italy; 2 Department of Oncology and Hemato-oncology, University of Milan, Milan, Italy

**Keywords:** lung resection, non-small cell lung cancer (NSCLC), perioperative treatments, radiotherapy, resectability, surgery

## Abstract

Resectability in non-small cell lung cancer (NSCLC) is shifting from a purely anatomic and surgical concept, based on technical feasibility of an R0 resection, to an integrated, multidisciplinary determination that incorporates tumor biology, expected benefit from perioperative systemic therapy and operative risk. Contemporary guidelines emphasize that resectability—particularly in stage III disease—should be defined by an experienced multidisciplinary team (MDT) with thoracic surgical input, alongside standardized staging and biomarker assessment to guide systemic options. Perioperative immune checkpoint inhibitor (ICI) plus chemotherapy regimens have expanded the population considered for curative-intent surgery by improving pathologic response and event-free survival, while also introducing new determinants of resectability, such as likelihood of completing multimodality therapy and managing treatment-related toxicity. Resectability in locally advanced (stage III) NSCLC is a multidisciplinary determination that complete oncologic resection is achievable with acceptable operative risk, and that surgery adds value within a multimodality plan rather than being based on anatomy alone. Key elements of resectable stage III NSCLC are: R0 resection goal with guideline-concordant nodal dissection; nodal extent drives resectability more than T stage as N3 is generally unresectable while single-station, non-bulky N2 is most commonly considered potentially resectable, whereas bulky and - or multistation N2 often argues against surgery, with persistent lack of consensus in the literature. The aim of this review is to focus on the key elements currently redefining the concept of resectability in the landscape of perioperative treatment for lung cancer, with particular regard to locally advanced disease, which represents the most challenging scenario for all professionals involved in the multidisciplinary management of lung cancer.

## 1 Introduction

Resectability in non–small-cell lung cancer (NSCLC) can no longer be framed as a purely anatomic and surgical question, because perioperative systemic therapies now change both the probability and the oncologic value of surgery across the traditional stage II–III boundary. Contemporary practice guidelines increasingly emphasize that resectable versus unresectable disease—particularly within stage III and N2 phenotypes—is a dynamic, multidisciplinary determination that must integrate surgical feasibility, anticipated systemic control, biomarker-defined treatment opportunities, and the competing curative-intent alternative of definitive chemo-radiotherapy (CRT) followed by consolidation immunotherapy (1, 2).

Over the past several years, randomized phase III trials have established neoadjuvant and perioperative chemo-immunotherapy as standards of care for many patients with operable, non-metastatic NSCLC, with consistent improvements in event-free survival (EFS) and substantial increases in pathological complete response (pCR), without compromising surgical feasibility (3, 4). These advances have shifted clinical decision-making from a binary “surgery first vs not” paradigm toward a sequence-planning problem: selecting the right preoperative regimen, anticipating technical complexity (e.g., fibrosis, nodal sterilization without radiographic shrinkage), deciding when post-induction resectability should be reassessed, and determining whether - and for whom - adjuvant immunotherapy adds value after an effective neoadjuvant course (5).

This evolution is most disruptive in stage III disease, where historic boundaries were largely defined by radiographic nodal burden (bulky or multistation N2), perceived likelihood of complete resection, and institutional comfort with trimodality care. Modern guidance acknowledges persistent equipoise in subsets—especially potentially resectable bulky or multistation N2—between definitive CRT strategies and surgery-inclusive approaches incorporating neoadjuvant/perioperative chemoimmunotherapy, reinforcing that resectability is inseparable from multidisciplinary team (MDT) expertise and local outcomes (6). In parallel, consensus statements increasingly codify process measures—mandatory thoracic surgical assessment before initiating induction therapy, standardized staging and restaging approaches, and the need for surgeons skilled in complex resections after immunotherapy—to ensure that expanding candidacy does not erode oncologic quality or patient safety (5).

At the same time, perioperative treatment has made tumor biology a pivotal factor in determining who should undergo surgery. Routine incorporation of at least EGFR and ALK testing, as well as PD-L1 when immunotherapy is contemplated, is now central because actionable driver alterations can redirect perioperative intent toward targeted adjuvant strategies and away from chemo-immunotherapy, while emerging translational tools such as circulating tumor DNA (ctDNA) kinetics and depth of pathological response are being positioned as potential decision modifiers for escalation or de-escalation after surgery (7). This creates a new conceptual framework: resectability is not simply whether surgery is technically possible today, but whether surgery remains the optimal curative component after considering how perioperative therapies can convert borderline disease to an R0 resection candidate (8).

This review therefore examines how perioperative immunotherapy and targeted therapy are redefining resectability in NSCLC, with a focus on (i) evolving anatomic and nodal criteria in the era of induction chemo-immunotherapy, (ii) practical surgical considerations and real-world feasibility after chemo-immunotherapy, (iii) biomarker-driven selection and postoperative management, and (iv) the remaining controversies where the field lacks direct comparative trials (neoadjuvant-only vs perioperative vs surgery-first with adjuvant strategies) and where standardized terminology for “resectable,” “borderline/potentially resectable,” and “unresectable” remains urgently needed.

## 2 Evolving anatomic and nodal criteria in the era of induction chemo-immunotherapy

**Figure 1:**
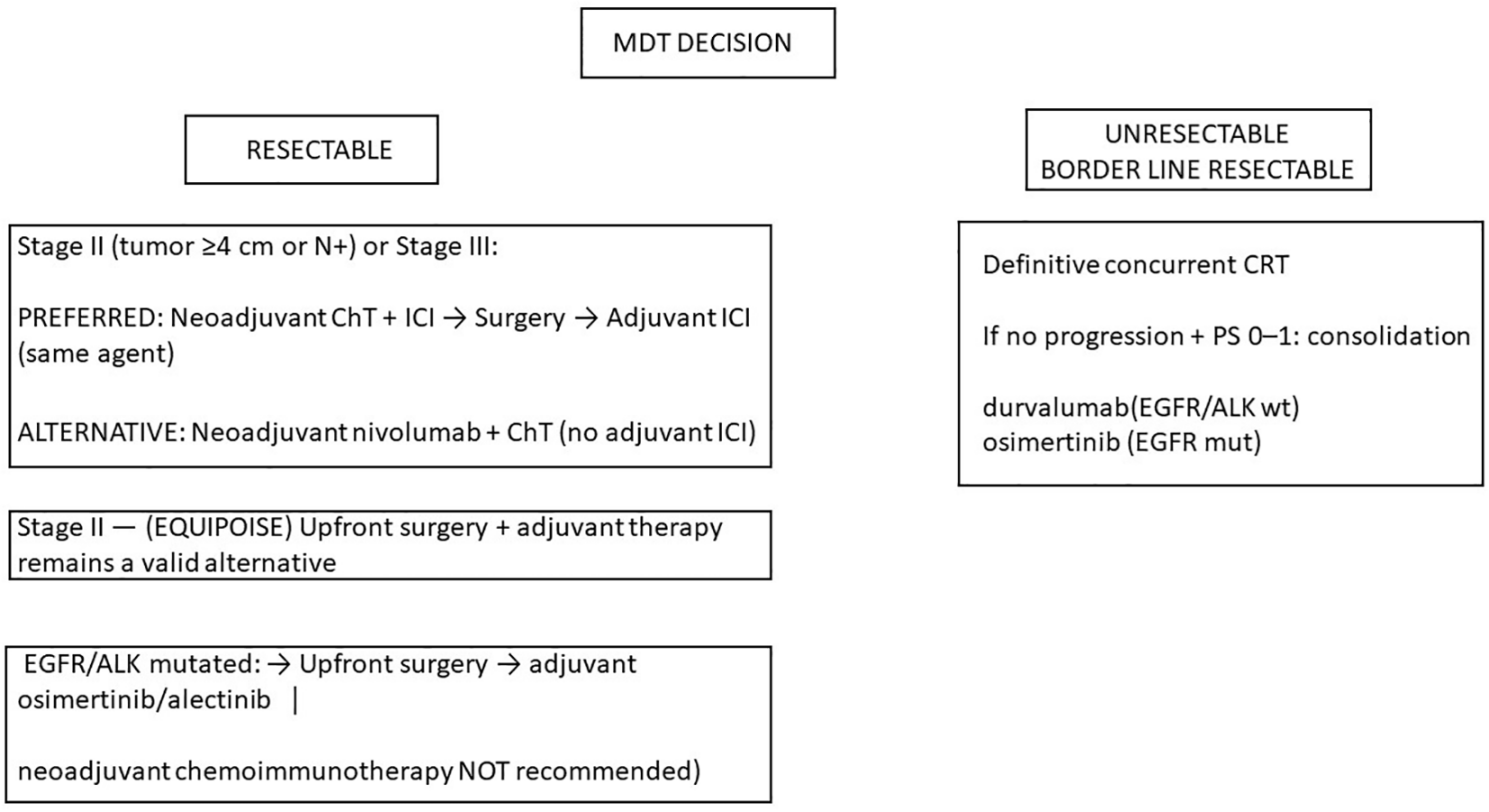
Decision algorithm for multidisciplinary resectability evaluation part A.

**Figure 2:**
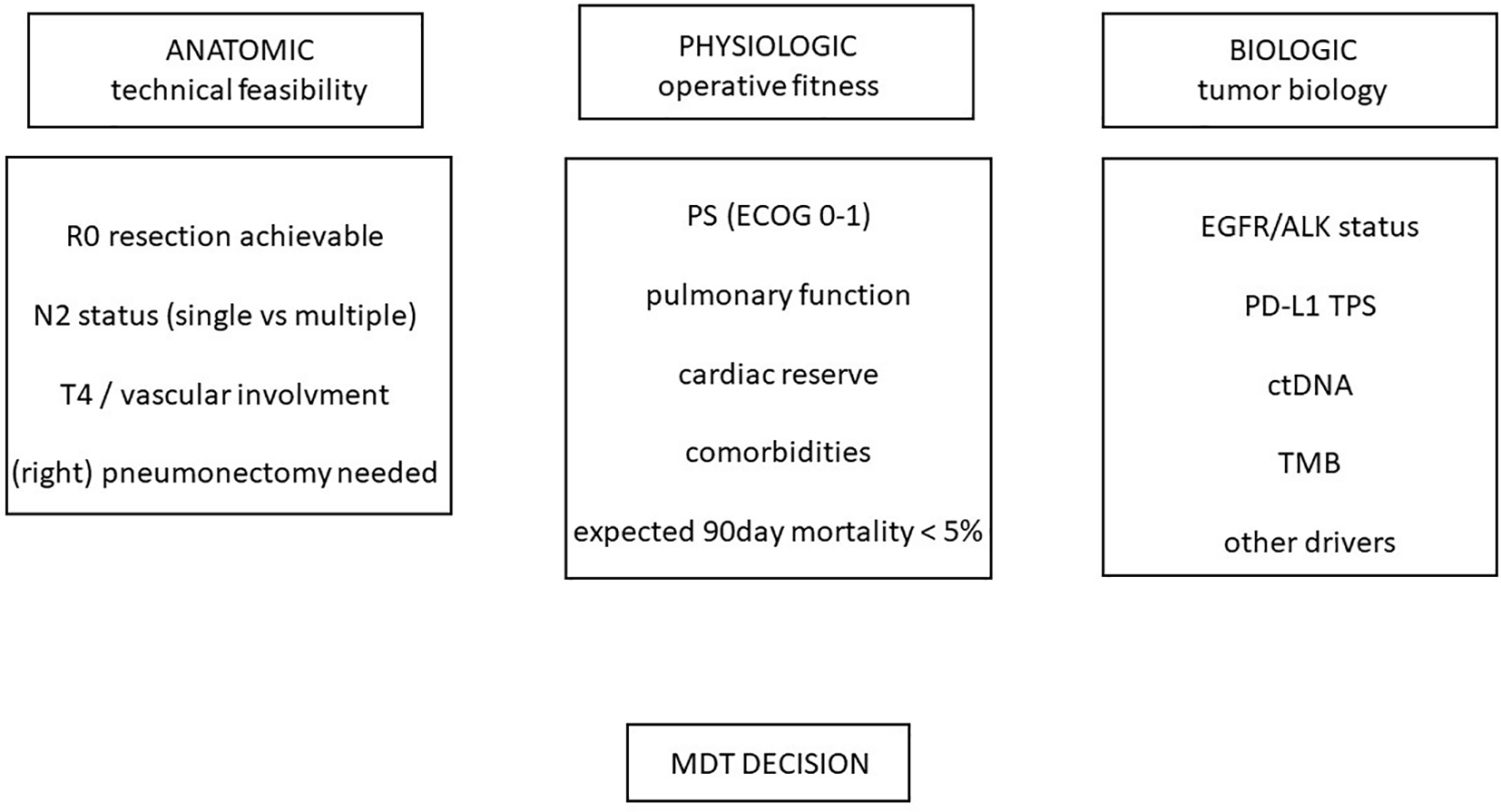
Decision algorithm for multidisciplinary resectability evaluation part B.

Effective induction therapy—particularly neoadjuvant and perioperative chemo-immunotherapy—has shifted resectability in NSCLC from a static, imaging-defined anatomic label to a dynamic, MDT- and surgeon-determined probability of achieving an oncologic complete resection with acceptable risk, even in select stage III phenotypes where nodal disease historically precluded surgery. Contemporary guidance identifies N2 disease as the key inflection point: ESMO recommends individualized MDT selection of neoadjuvant and perioperative systemic therapy versus definitive concurrent CRT for N2 presentations, notes persistent uncertainty for bulky or multistation N2, and endorses structured resectability frameworks that generally consider N3 unresectable while supporting surgery-inclusive multimodality strategies in carefully defined IIIA and selected IIIB subsets (1). In parallel, the biologic efficacy of induction chemo-IO is altering interpretation of nodal burden: CheckMate 816 demonstrated that adding nivolumab to platinum chemotherapy in resectable, node-positive or ≥4 cm tumors markedly increases pathologic response and improves event-free survival, supporting broader use of induction strategies to enable curative-intent resection in node-positive disease when R0 remains feasible (1, 3). ASCO’s stage III guideline reinforces that surgery after induction is appropriate only for selected IIIA(N2) patients when R0 is achievable, N3 is excluded by multidisciplinary consensus, and operative mortality is acceptably low, underscoring that nodal resectability depends on rigorous invasive mediastinal staging and center/surgeon outcomes rather than CT and PET appearances alone (9). IASLC consensus recommendations further operationalize this evolution by advising that, after neoadjuvant chemo-IO, CT reassessment is required but routine invasive mediastinal restaging is not, and that patients without progression who remain resectable should proceed to surgery—recognizing that post-induction nodal sterilization and fibrosis may decouple radiographic nodal size from pathologic status and should not automatically preclude resection (5). Finally, emerging prospective “conversion” experiences in initially unresectable stage III define unresectability largely by anatomic and nodal features and use MDT reassessment after immunotherapy-based induction to select patients for radical resection, illustrating how modern induction regimens are expanding traditional anatomic and nodal boundaries while remaining dependent on expert MDT judgment and meticulous surgical technique rather than universally standardized size- or station-based cutoffs (**Figures 1**, **2**).

## 3 Practical surgical considerations and real-world feasibility after chemo-immunotherapy

Chemo-immunotherapy is increasingly used in the neoadjuvant and perioperative management of NSCLC and has forced surgeons and MDTs to re-evaluate resectability beyond anatomic criteria alone. In real-world practice, feasibility depends on whether the patient remains operable after systemic therapy, whether an R0 resection remains technically achievable despite therapy-induced hilar and mediastinal changes, and whether perioperative risks are acceptable within predefined treatment windows (4). Post chemo-immunotherapy resectability is best viewed as dynamic and conditional, integrating oncologic resectability, physiologic operability, and timing feasibility.

Oncologic resectability is defined as the probability of R0 resection with adequate margins and nodal clearance given post-treatment anatomy and residual disease distribution. Physiologic operability depends on cardiopulmonary reserve after induction therapy, including deconditioning, anemia, thromboembolic events, or immune-related toxicities. Timing feasibility reflects the ability to proceed to surgery before fibrosis consolidates and before delays compromise systemic therapy sequencing (1, 4).

Pre-treatment “borderline resectable” disease should be discussed with explicit contingencies such as conversion to definitive chemo-radiation, altered extent of resection, or abandonment if oncologic or physiologic parameters deteriorate.

With regard to restaging after chemo-immunotherapy, radiographic response may underestimate or misrepresent pathologic response because of immune-cell infiltration, oedema, or necrosis. CT and PET findings must therefore be interpreted cautiously, as metabolic response may lag or be confounded by inflammation. Nodal reassessment remains critical because persistent FDG uptake may be inflammatory, while radiographically normalized nodes can still harbor viable tumor. For this reason, invasive mediastinal restaging is often favored when management depends on nodal sterilization (e.g., baseline N2 disease), although fibrosis and necrosis can reduce sampling yield and complicate EBUS or mediastinoscopy (11, 12). Given these treatment-induced changes, institutions should define upfront what constitutes sufficient downstaging and apply a consistent algorithm to minimize subjective decision-making.

From a technical perspective, chemo-immunotherapy can induce dense hilar inflammation, obliterate tissue planes, and cause nodal adhesions, significantly affecting surgical feasibility. Sticky lymph nodes and perivascular fibrosis may increase the risk of pulmonary artery injury and prolong operative time. Prior cytotoxic exposure and immune-mediated tissue changes may also increase bleeding risk and complicate vascular control. Complete nodal dissection can become more challenging, although systematic nodal evaluation remains essential for staging and postoperative planning. We therefore recommend early planning for proximal vascular control, availability of open surgical instruments and vascular reconstruction capabilities, and preoperative counselling regarding the increased likelihood of conversion to an open approach (13).

Many centers attempt VATS or robotic approaches following induction therapy; however, feasibility depends on the initial disease burden, tumor centrality, and extent of nodal involvement. A low threshold for conversion is warranted when dissection becomes unsafe because of bleeding, loss of anatomical planes, or inability to achieve adequate oncologic margins. Conversion from a minimally invasive to an open approach should be viewed as a safety strategy rather than a complication. Central tumors treated after induction therapy—particularly those associated with bulky baseline N1 or N2 disease—often require preparedness for thoracotomy from the outset (13).

Induction chemo-immunotherapy can convert previously unresectable patients into surgical candidates; however, it may also increase the complexity of subsequent resections. When feasible, parenchyma-sparing sleeve resections should be favored over pneumonectomy to reduce morbidity and preserve pulmonary function. Nevertheless, treatment-related inflammation and fibrosis can make airway dissection and bronchial reconstruction substantially more challenging. Pneumonectomy after induction therapy remains high risk, particularly for right-sided resections and patients with limited cardiopulmonary reserve. Similarly, chest wall or vascular involvement requires careful reassessment because radiographic regression does not necessarily correlate with safe intraoperative dissection from major vascular structures.

Accordingly, centers managing complex cases should anticipate the potential need for bronchoplasty or angioplasty, ensure the availability of vascular clamps and reconstruction materials, and consider a dual-attending surgeon strategy for procedures at high risk of conversion or major vascular complications (13).

With regard to perioperative safety, a unique challenge associated with immunotherapy is the occurrence of unpredictable immune-related adverse events (irAEs), which may transform an otherwise operable patient into a high-risk surgical candidate. Pneumonitis is of particular concern, as it may be subclinical before surgery yet become clinically significant following pulmonary resection. Active screening and a low threshold for postponing surgery are therefore warranted when pneumonitis is suspected. Although less common, myocarditis, pericarditis, and arrhythmias are associated with substantial morbidity and mortality and require prompt evaluation when symptoms, troponin elevation, or electrocardiographic abnormalities are detected. Other irAEs, including hepatitis, colitis, adrenal insufficiency, and thyroiditis, may adversely affect perioperative stability and complicate corticosteroid management. Close multidisciplinary coordination with the treating oncologist is therefore essential. The interval between the last treatment infusion and surgery should balance recovery from treatment-related toxicities against the risks of prolonged delays, including disease progression, fibrosis, and increasing technical complexity (10, 13).

Before surgery, adequate hematologic recovery, nutritional status, and functional capacity should be confirmed. Surgery should generally be deferred in the presence of active grade ≥2 irAEs unless urgent intervention is required. Close coordination among surgical, medical oncology, and perioperative teams is also necessary to ensure surgery occurs within the predefined institutional timeframe established by the neoadjuvant protocol, minimizing delays and maintaining adherence to evidence-based pathways (10, 13).

High complexity resections after neoadjuvant chemo-IO for resectable NSCLC are feasible and generally not associated with an excess of perioperative mortality, but they cluster in centrally located tumors and/or post-treatment nodal “fibro-inflammatory” changes, which can force bronchoplastic/vascular procedures, increase thoracotomy and conversion rates, and occasionally reveal new carinal involvement at bronchoscopy despite stable imaging. Thoracic surgeons planning to operate after neoadjuvant chemoimmunotherapy should be skilled in advanced pulmonary surgical maneuvers (e.g., bronchoplasty/sleeve techniques and vascular reconstruction) and cases with uncertain feasibility should be discussed in a multidisciplinary tumor board before proceeding to surgery (5).

Direct institutional volume–outcomes data specific to *post–chemo-IO* resections in resectable NSCLC are limited, so practice is currently guided mainly by broader thoracic surgery volume/specialization literature plus expert consensus to centralize complex post-neoadjuvant cases and ensure availability of advanced reconstructive skills.

## 4 Biomarker-driven selection and postoperative management

Biomarker-driven selection in locally advanced NSCLC starts with the premise that patients should not be committed to a perioperative plan until oncogenic drivers (at least EGFR ex19del/L858R and ALK fusions) and PD-L1 tumor proportion score are known, as these determine immunotherapy suitability and the role of targeted postoperative therapy. ESMO and other expert groups emphasize early molecular testing, often on the diagnostic biopsy, to avoid delays and unnecessary exposure of EGFR- or ALK-driven tumors to PD-(L)1–based strategies that are not preferred in the curative-intent setting (1, 5).

Once resectability is established, biomarker results define the systemic therapy backbone. In tumors harboring EGFR ex19del/L858R, adjuvant osimertinib is the postoperative cornerstone, typically following stage-appropriate platinum-based chemotherapy when indicated, and perioperative chemo-immunotherapy is generally avoided. ESMO explicitly recommends adjuvant osimertinib after complete resection, while ASCO similarly supports its use following definitive local therapy in appropriate patients (1, 9). Likewise, identification of an ALK fusion shifts postoperative management toward adjuvant alectinib, integrated with chemotherapy according to stage and patient fitness, with immunotherapy-based perioperative approaches generally not preferred (1).

For EGFR/ALK wild-type tumors, immunotherapy becomes a key option and PD-L1 expression helps refine postoperative treatment. In resected stage II–IIIA disease treated with adjuvant platinum chemotherapy, ESMO supports adjuvant atezolizumab for PD-L1–positive tumors and pembrolizumab as an option irrespective of PD-L1 status in eligible patients. NCCN also considers perioperative chemo-immunotherapy regimens preferred for selected resectable stage II–III disease, provided EGFR and ALK status are established upfront because they directly influence eligibility and subsequent management (1, 2).

Postoperative care should be viewed as a continuous pathway rather than discrete steps. Surgical-pathologic factors determining recurrence risk and local control requirements (margin status, pN category, and nodal burden) should first be assessed, followed by biomarker-guided systemic therapy, with postoperative radiotherapy reserved for specific indications. ESMO advises against routine PORT after R0 resection, whereas PORT may be considered after R1 resection following multidisciplinary discussion. ASCO similarly does not recommend routine PORT after complete resection in many N2 scenarios, emphasizing that locally advanced disease alone does not mandate radiotherapy (1, 9).

For unresectable stage III disease treated with definitive CRT, biomarker-driven management takes the form of post-CRT consolidation. Here too, genotype is decisive: ASCO recommends consolidation osimertinib for tumors with EGFR ex19del/L858R after CRT without progression, whereas ESMO continues to recommend durvalumab consolidation for EGFR–wild-type disease (1, 9).

## 5 Remaining controversies

The central unresolved issue is the choice among three different sequencing philosophies for resectable stage II–III NSCLC—neoadjuvant-only immunochemotherapy, perioperative immunochemotherapy (neoadjuvant plus planned adjuvant), and surgery-first followed by adjuvant systemic therapy—without direct randomized trials that compare these strategies against each other in the same contemporary population, so most conclusions are necessarily indirect and contingent on trial design differences. This limitation is explicitly acknowledged in ESMO, IASLC consensus recommendations, and NCCN Insights, which all emphasize that current evidence is derived from studies built around different control arms and different types of therapy rather than clean head-to-head sequencing comparisons (1, 5). Practically, the field’s debate often comes down to whether the value of immunotherapy in curative-intent disease is mostly captured *before* surgery (by shrinking the tumor, sterilizing micrometastases early, and increasing the chance of pathologic response), or whether it’s crucial to continue immunotherapy *after* surgery to maintain systemic pressure on residual disease. The perioperative phase III programs—KEYNOTE-671 (pembrolizumab), AEGEAN (durvalumab), and CheckMate 77T (nivolumab)—demonstrate that a combined neoadjuvant + adjuvant approach improves event-free survival versus control regimens that do not include the full perioperative immunotherapy package, which is why perioperative approaches have moved quickly into guidelines (4, 14, 15). However, because these trials typically test a bundle (neoadjuvant chemo-ICI plus adjuvant ICI) against a control that doesn’t contain that bundle, they do not disclose the specific, incremental contribution of the adjuvant immunotherapy component when the patient has already received effective neoadjuvant chemo-ICI and undergone complete resection. That uncertainty is why IASLC and NCCN describe the evidence gap in direct comparisons, and why some groups lean on indirect comparisons—such as a 2025 network meta-analysis—while still acknowledging that network results can’t substitute for a randomized “with vs without adjuvant ICI” design because of cross-trial heterogeneity (2, 5, 16).

A closely related controversy is how to interpret strong perioperative results in the context of the modern “surgery-first” pathway, which has itself evolved: many patients now receive adjuvant chemotherapy, and some are candidates for adjuvant immunotherapy depending on PD-L1 and biomarker status. Most perioperative trials were not designed to answer the question, “Is perioperative immunochemotherapy superior to optimal surgery-first with fully delivered, guideline-concordant adjuvant therapy?” Instead, they answer, “Is adding perioperative immunotherapy to a particular trial backbone better than that trial backbone alone?” ESMO and IASLC are explicit that this mismatch in comparators is a major reason sequencing remains debated even as multiple positive trials accumulate (1, 5).

Another major unresolved area is how to individualize postoperative management after neoadjuvant or perioperative immunochemotherapy using response-adaptive markers. Clinicians naturally want to use pathologic pCR or major pathologic response (MPR) as a switch—stop adjuvant immunotherapy if the resection specimen shows deep response or escalate if there is substantial residual viable tumor—but none of the pivotal phase III perioperative trials were built to randomize patients based *on* pathologic response into de-escalation versus continuation, or escalation versus standard completion. The exploratory nodal-status analyses (for example in CheckMate 77T) are helpful for hypothesis generation about who benefits most, but they still don’t resolve the core “response-adapted” sequencing question because they are not randomization strata with treatment strategy assignment (17).

Stage and risk subgrouping adds another layer of uncertainty, especially the question of whether stage II disease should default to the same aggressive perioperative paradigm as bulkier, multi-station N2 stage III disease. IASLC highlights ongoing equipoise in parts of stage II, whereas many clinicians feel the highest-risk stage III phenotypes are where early systemic therapy is most compelling—yet, again, we lack direct comparative trials that randomize stage II–specific populations to surgery-first/adjuvant versus neoadjuvant-only versus perioperative, using the same systemic agents (5).

Maturing data now show an overall survival (OS) benefit for neoadjuvant chemo-immunotherapy vs neoadjuvant chemotherapy alone, with the most mature and definitive OS signal coming from CheckMate 816 (3), and an emerging OS benefit in the perioperative pembrolizumab strategy (KEYNOTE-671) (14).

Finally, even assuming that perioperative therapy is best on average, there are still unanswered pragmatic questions that only direct strategy trials can settle: whether longer perioperative exposure meaningfully increases immune-related toxicity or interferes with timely surgery in a way that offsets benefits, whether completion rates and real-world adherence tilt the balance toward neoadjuvant-only approaches, and how best to integrate postoperative radiation or additional systemic therapy when margins are close/positive or when the pathologic stage remains high after induction. These are the kinds of real-world sequencing trade-offs that ESMO and multidisciplinary consensus documents flag as unresolved precisely because existing trials were not structured to compare strategy packages head-to-head (1, 5, 6).

## 6 Conclusion

Resectability in contemporary lung cancer care should be redefined as a dynamic, multidisciplinary, biology-informed construct rather than a static anatomic label (18). The rapid integration of perioperative systemic treatments—especially neoadjuvant chemoimmunotherapy—has expanded the population that can realistically achieve curative-intent outcomes, while simultaneously raising the stakes for precise selection, timing, and surgical execution. Across current standards, surgery remains the cornerstone for fit patients with early-stage and selected locally advanced NSCLC, but the decision to operate increasingly depends on whether an R0 resection is achievable with acceptable perioperative risk and whether alternative definitive approaches (notably concurrent chemoradiotherapy with consolidation immunotherapy) offer a more favorable risk–benefit profile for a given stage III presentation (19, 20). These comparisons are particularly consequential in borderline scenarios such as multistation or bulky N2 disease, where global practice variation persists and where “resectable” must incorporate institutional expertise, anticipated extent of resection (lobectomy vs pneumonectomy), and the patient’s physiologic reserve and preferences.
